# LncRNA FAM13A-AS1 Promotes Renal Carcinoma Tumorigenesis Through Sponging miR-141-3p to Upregulate NEK6 Expression

**DOI:** 10.3389/fmolb.2022.738711

**Published:** 2022-03-23

**Authors:** Xin Jun Wang, Si Li, Jiang Fang, Zhi Jian Yan, Guang Cheng Luo

**Affiliations:** ^1^ Department of Urology, Zhongshan Hospital Xiamen University, School of Medicine, Xiamen University, Xiamen, China; ^2^ The Third Clinical Medical College, Fujian Medical University, Fuzhou, China

**Keywords:** FAM13A-AS1, miR-141-3p, NEK6, prognostic markers, therapeutic targets, rcc

## Abstract

Long non-coding RNAs are a diverse catalog of RNAs that have been implicated in various aspects of tumorigenesis. Emerging evidence indicates that they play crucial roles in tumor growth, disease progression, and drug resistance. However, the clinical significance of lncRNAs in tumor behavior prediction and disease prognosis as well as the underlying mechanism in renal cell carcinoma (RCC) remains elusive. By analyzing the gene expression profiles of 539 RCC patients from the TCGA cohort and 40 RCC patients from an independent cohort, we identified FAM13A-AS1, a poorly studied lncRNA, upregulated in RCC patients. Knockdown experiments revealed that FAM13A-AS1 promotes cell proliferation, migration, and invasion by interacting with miR-141-3p. FAM13A-AS1 regulates the expression of NEK6 by decoying miR-141-3p. In addition, there was a strong positive correlation between the expression of FAM13A-AS1 and NEK6 in RCC patients. In summary, our results demonstrate the oncogenic role of FAM13A-AS1 in RCC and suggest that it promotes tumorigenesis by upregulating the expression of NEK6 by competitively binding to miR-141-3p.

## Introduction

Kidney cancer is among the 10 most common cancers in both men and women, representing 4% of all new cancer cases annually in United States (Escudier et al., 2019). Renal cell carcinoma (RCC) is the most dominant form of kidney cancer, accounting for up to 85% of all cases. RCC has the highest mortality rate of genitourinary cancers, and the incidence of RCC has risen steadily, especially in developed countries. It has been reported that approximately 295,000 new kidney cancer cases are diagnosed, and approximately 134,000 deaths are recorded worldwide (Hsieh et al., 2017; Escudier et al., 2019). Although the 5-year survival rate has recently increased, it is still ranked as the seventh leading cause of mortality in the United States (Jemal et al., 2007). Localized RCC can be treated with surgical resection, radiation, or local ablation. Despite advances in the curative treatment, more than 30% of patients would eventually develop metastases, requiring more complicated therapy and low mortality. The likelihood of successful treatment relied heavily on the early diagnosis (Barata and Rini, 2017). However, early clinical manifestations of RCC are diverse and may give rise to a range of non-specific and often misattributed symptoms. Therefore, developing better models for risk assessment, diagnosis, and prognosis has become an urgent need for managing RCC, requiring a fundamental understanding of the disease’s molecular mechanisms.

Once dismissed as “junk DNA,” lncRNAs are now implicated in various fundamental biological processes and disease development (Kopp and Mendell, 2018). Evidence accumulated over the past decade has revealed that lncRNAs function in diverse mechanisms, such as signals, RNA decay, miRNA sponging, guides, and RNA scaffolding. Notably, a growing number of lncRNAs are emerging as master regulators of cancer progression and tumor metastasis (Slack and Chinnaiyan, 2019). Some examples include HOTAIR (Gupta et al., 2010; Alves et al., 2013; Kim et al., 2013), FILNC1 (Xiao et al., 2017), and GAS5 (Zhang et al., 2017). Dysregulation of these genes is associated with renal tumor metabolism, pancreatic cancer metastasis, and prostate cancer proliferation.

In this study, we identified an RCC-associated lncRNA, FAM13A-AS1, through gene expression analysis on a cohort of 539 TCGA-KIRC datasets, whose function in oncogenesis has not yet been investigated. We found that FAM13A-AS1 expression was increased in both primary tumor and RCC cell lines. Elevated expression of FAM13A-AS1 is associated with poor prognosis in patients with RCC. We further demonstrated that FAM13A-AS1 promotes cell proliferation, migration, and invasion by targeting NEK6 when acting as a sponge for miR-141-3p. Thus, our study identified the FAM13A-AS1/miR-141-3p/NEK6 axis in prostate tumorigenesis and indicated them as promising prognostic markers and potential therapeutic targets for RCC patients.

## Methods

### Patient Samples

RCC samples (*n* = 40) and their paired normal tissues (*n* = 40) were obtained from the Department of Urology, Zhongshan Hospital, Xiamen University (Xiamen, China). Informed consent was obtained from all patients who were without preoperative treatments before surgery, and the study was approved by the Institutional Review Board of Xiamen University.

### Cell Culture

293 T cells were purchased from IMMOCELL (catalog number: IM-H222, Xiamen, China). The human kidney proximal tubule epithelial cell line HK-2 and malignant cancer cell lines 786-O, A-498, SN12-PM6, and Caki-1, were purchased from American Type Culture Collection (ATCC, Manassas, VA, United States). The authenticity of these cell lines was confirmed using STR profiling analysis. The cells were cultured in a medium consisting of 90% DMEM (Gibco, catalog number: 11965092, Shanghai, China) supplemented with 10% FBS (Gibco, catalog number: 12483020, Shanghai, China) in an incubator at 37 °C and 5% CO_2._

### Plasmid and Lentiviral Transduction

The human FAM13-AS1 fragment or human NEK6 3′ UTR containing the putative binding sites targeted by miR-141-3p were cloned into a pmirGLO vector (Promega, Madison, WI, United States), which were named as FAM13-AS1 WT and NEK6 3′ UTR WT, respectively. The mutant FAM13-AS1 or mutant NEK6-3′ UTR was also cloned into a pmirGLO vector (Promega, Madison, WI, United States), which were named as FAM13-AS1 MUT and NEK6 3′ UTR MUT, respectively.

For RNAi, short hairpin RNAs targeting FAM13-AS1 and its negative control were constructed using the pLKO.1-TRC vector (Anithela, Xiamen, Fujian, China). miR-141-3p mimics and inhibitors (Table 1) were purchased from Guangzhou RiboBio Co., Ltd, and the transfection was carried out using Lipofectamine™ 2000 Transfection Reagent (Invitrogen, catalog number: 11668500, Shanghai, China) following the manufacturer’s instructions. For the expression of NEK6 in cells, the coding sequence of NEK6 was cloned into the PCDH-EF1a-mcs-T2A-puro vector (Anithela, Xiamen, Fujian, China). Cloning was performed using the ClonExpress Ultra One Step Cloning Kit (Vaztme, C115-01, Nanjing, China), and the relevant primers are listed in Table 2.

**TABLE 1:** Sequences of mimics and inhibitors.

Mimic NC	5′-uug​uac​uac​aca​aaa​gua​cug-3′
miR-141-3p mimic	5′-uaa​cac​ugu​cug​gua​aag​aug​g-3′
inhibitor NC	5′-cag​uac​uuu​ugu​gua​gua​caa-3′
miR-141-3p inhibitor	5′-cca​ucu​uua​cca​gac​agu​guu​a-3′

**TABLE 2:** Primers for plasmid construction.

pmirGLO-NEK6 3′ UTR WT-F	5′-gag​ctc​gct​agc​ctc​gag​gtg​tgc​tta​att​tac​tct​g-3′
pmirGLO-NEK6 3′ UTR WT-R	5′-cat​gcc​tgc​agg​tcg​acg​tac​att​aat​tct​cga​ctt​c-3′
pmirGLO-NEK6 3′ UTR MUT-F	5′-cat​gat​cgc​aac​gaa​gca​ttt​tcc​tcc​atg​g-3′
pmirGLO-NEK6 3′ UTR MUT-R	5′-aat​gct​tcg​ttg​cga​tca​tga​aat​gaa​ccg​c-3′
pmirGLO-FAM13A-AS1 WT-F	5′-ctc​gct​agc​ctc​gag​tga​aaa​ctt​tca​gat​gct​tc-3′
pmirGLO-FAM13A-AS1 WT-R	5′-cat​gcc​tgc​agg​tcg​aca​act​ttg​tca​ttt​aaa​ata​tat​tg-3′
pmirGLO-FAM13A-AS1 MUT-F	5′-ctt​gag​caa​cga​gga​gga​act​tga​gtc-3′
pmirGLO-FAM13A-AS1 MUT-R	5′-ctc​ctc​gtt​gct​caa​gaa​ggt​ttt​tca​c-3′
pLKO.1-shFAM13A-AS1-F	5′-ccg​gaa​ctt​tca​gat​gct​tct​tca​ttg​ctc​gag​caa​tga​aga​agc​atc​tga​aag​ttt​ttt​t-3′
pLKO.1-shFAM13A-AS1-R	5′-aat​taa​aaa​aac​ttt​cag​atg​ctt​ctt​cat​tgc​tcg​agc​aat​gaa​gaa​gca​tct​gaa​agt​t-3′
pCDH-NEK6-F	5′-gct​agc​gaa​ttc​gcc​acc​atg​gag​gcc​act​gga​tgg​g-3′
pCDH-NEK6-R	5′-tca​gcg​gcc​gcg​gat​ccg​gtg​ctg​gac​atc​cag​atg-3′

Plasmid shFAM13-AS1 was co-transferred with pMD2G and pspax2 into 293 T cells to package the lentivirus. After 72 h, the supernatants containing lentiviruses were collected. Two days after lentiviral infection, 786-O cells were cultured in the presence of 1.0 μg/ml of puromycin (Sigma, San Francisco, CA, United States) for 8 days, which was named as shFAM13-AS1 cell.

### RNA Extraction, Reverse Transcription and RT-qPCR

Prostate cancer cell lines, transfected cells, and tissue homogenates were harvested for RNA extraction. Total RNA was isolated using TRIzol reagent (Invitrogen, catalog number: 15596018, Grand Island, NY, United States) according to the manufacturer’s instructions, and 1 μg RNA was reverse transcribed into cDNA using the Superscript II First-Strand Synthesis System (Invitrogen, catalog number: 18080051, Shanghai, China). Primers used for RT-qPCR are listed in Table 3.

**TABLE 3:** Primers for RT-qPCR.

U6-RT	5′-aac​gct​tca​cga​att​tgc​gt-3′
U6-F	5′-ctc​gct​tcg​gca​gca​ca-3′
U6-R	5′-aac​gct​tca​cga​att​tgc​gt-3′
18S-F	5′-agg​cgc​gca​aat​tac​cca​atc​c-3′
18S-R	5′-gcc​ctc​caa​ttg​ttc​ctc​gtt​aag-3′
miR-141-3p-RT	5′-gtc​gta​tcc​agt​gca​ggg​tcc​gag​gta​ttc​gca​ctg​gat​acg​acc​cat​ct-3′
miR-141-3p-F	5′-gcg​cgt​aac​act​gtc​tgg​taa-3′
miR-141-3p-R	5′-gag​gta​gag​aat​aga​atg​ata​g-3′
FAM13A-AS1-F	5′-agt​gtt​agg​agg​aac​ttg​ag-3′
FAM13A-AS1-R	5′-gct​ggg​caa​ata​ctc​tga-3′
NEK6-F	5′-atc​cat​gag​aac​ggc​tac​aa-3′
NEK6-R	5′-tct​ggc​aca​ggg​aga​aga​g-3′

### Western Blotting

Cells and tissue homogenates were harvested and lysed in RIPA buffer (50 mM Tris-HCl [pH 7.4], 1% Nonidet P-40, 0.25% sodium deoxycholate, 150 mM NaCl, 1 mM EDTA, 1 mM PMSF, 1 mg/ml aprotinin, 1 mg/ml leupeptin, 1 mg/ml pepstatin, 1 mM Na_3_VO_4_, and 1 mM NaF). The protein concentration in the lysate was quantified using the BCA Kit. The cell lysates were loaded onto an SDS-PAGE gel for electrophoresis. After transfer of the proteins onto a piece of nitrocellulose membrane, targets were detected by western blotting using primary antibodies, including NEK6 antibody (1 : 2000, catalog number: 10378-1-AP, Proteintech, Wuhan, China), GAPDH antibody (1 : 10,000, catalog number: 10494-1-AP, Proteintech), secondary antibody, HRP-conjugated goat anti-mouse IgG (H + L) (1:10,000, catalog number: ab205719, Abcam, United States), HRP-conjugated goat anti-rabbit IgG (H + L) (1:10,000, catalog number: ab205718, Abcam), and an ECL Chemiluminescent Substrate Reagent Kit (Invitrogen, catalog number: WP20005, Shanghai, China). Protein expression levels were quantified using ImageJ software. The experiments were performed in triplicate.

### MTT Assay

After seeding into 96-well plates at a density of 10^3^ cells/well and cultured overnight, the cells were treated as indicated. Subsequently, the CyQUANT™ MTT Cell Viability Assay (Invitrogen, catalog number: V13154, Shanghai, China) was used to detect cell proliferation according to the manufacturer’s instructions.

### Invasion and Migration Assay

After transfection for 24 h, 3 × 10^5^ cells were seeded into the upper chambers of the transwell plates with and without matrigel for invasion and migration assays, respectively. After incubation in a serum-free medium for 24 h, the cells in the upper chambers were wiped away. The cells at the bottom of the upper chamber were fixed and stained with 4% paraformaldehyde and 0.5% crystal violet. The upper chamber was then dipped into distilled water to remove excess crystal violet. Migrated or invasive cells were counted under a microscope. The experiments were performed in triplicate.

### Luciferase Reporter Assay

Plasmids, FAM13-AS1 WT, FAM13-AS1 MUT, NEK6 3′-UTR WT, and NEK6 3′-UTR MUT were co-transfected into kidney cancer cells with miR-141-3p mimics or the negative control (mimic NC). After 48 h, the relative luciferase activity was measured with the ONE-Glo luciferase Assay System (Proteintech, catalog number: E6110, Wuhan, China) according to the manufacturer’s instructions.

### Analysis of the Cell Cycle

786-O or SN2-PM6 cells transfected with various plasmids were seeded at a density of 3×10^5^ cells/well in 6-well plates in a medium containing 90% DMEM plus 10% FBS. Upon receiving the desired treatments, the cells were trypsinized and fixed with 70% (v/v) ethanol at 4°^◦^C overnight. The fixed cells were stained with 50 μg/ml propidium iodide (PI) for 30 min at 37°C in the dark for cell cycle analysis. After incubation, the cells were analyzed using a FACScan flow cytometer (Beckman Coulter, United States) within 15 min. The fixed cells were stained with Annexin V-FITC and PI double staining solution for apoptosis analysis before being subjected to flow cytometry. The percentage of cells in each phase (G1, S, and G2/M) of the cell cycle and the percentage of cells undergoing apoptosis were determined using the Cell Quest software. The proliferation indexes were calculated using the following formula: PI=(S + G2/M)/(G1+S + G2/M).

### Fluorescence *in Situ* Hybridization (FISH)

Cells were seeded and grown overnight on glass coverslips. After immobilization and permeabilization, the cells were subjected to hybridization with 20 μM Cy3-labeled FAM13A-AS1, 18S, or U6 FISH probes. The nuclei of the cells were stained with 6-diamidino-2-phenylindole (DAPI). All images were captured using a confocal laser-scanning microscope.

### Animal Model

All animal experiments were approved by Institutional Review Board of ZhongShan Hospital, Xiamen University. Nude mice were purchased from Shanghai Lab at the Animal Research Center (Shanghai, China). Each mouse was injected with 5×10^6^ shFAM13-AS1 cells or negative control cells in the axilla. The long diameter 1) and short diameter 2) of tumors were measured every 4 days. The tumor volume (V) was calculated using formula V = ab (Hsieh et al., 2017)/2. The tumor was monitored for 38 days before the mice were sacrificed to dissect the tumor tissues. The tumor tissues were weighed and then ground using a grinder Tissuelyser-24 (Jingxin, Shanghai, China). The tissue homogenates were subjected to analyses of RT-qPCR and western blotting.

### TCGA Data Processing

Gene expression and RCC patient survival data were downloaded from the GDC website (https://portal.gdc.cancer.gov/). The TCGA database provides multiple types of data for 539 RCC cases. The expression value of FAM13-AS1 was classified as either high or low. Survival probability was calculated in days from the date of diagnosis to the time of death.

### Statistical Analysis

All image data were analyzed using GraphPad Prism 5 software and represented as mean ± standard deviation (SD). The Shapiro-Wilk test was performed to determine whether the data follows a normal distribution. The Levene’s test was used to ensure the homogeneity of variance. ANOVA followed by Tukey’s post-hoc test was used for multiple comparisons among three groups. Unpaired and paired student’ t-test were performed to compare the difference between two Unpaired and paired groups, respectively. Survival curves were calculated using the Kaplan-Meier method, and the significance was determined by the log-rank test. A Pearson correlation test was performed to assess the correlation among FAM13A-AS1, miR-141-3p, and NEK6 mRNA expression using software environment R, version 3.3.2. All results were considered statistically significant at *p* < 0.05.

## Results

### Expression of lncRNA FAM13-AS1 Is Upregulated and Associated With Poor Clinical Outcomes in RCC Patients

**FIGURE 1:**
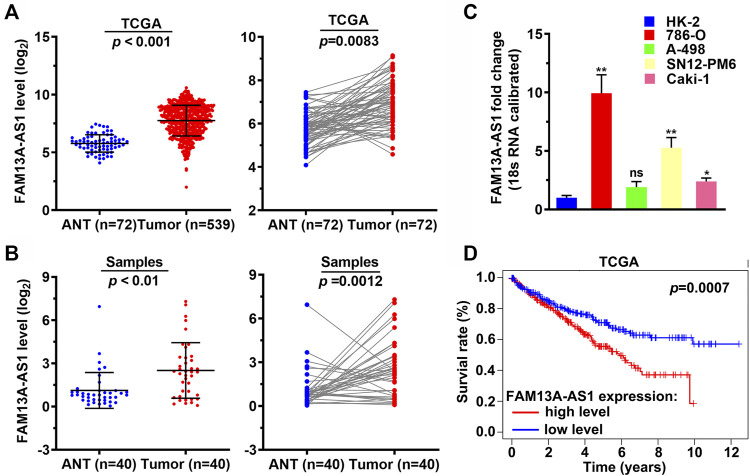
FAM13A-AS1 is upregulated in RCC and associated with poor clinical outcome. **(A)** Scatter plots showing the expression levels of FAM13A-AS1 in RCC (*n* = 539) and normal tissues (*n* = 72) from TCGA cohort. **(B)** Scatter plots showing the expression levels of FAM13A-AS1 in RCC (*n* = 40) and normal tissues (*n* = 40) from our cohort. **(C)** Bar plot showing the fold change in FAM13A-AS1 expression in human kidney epithelia and cancer cell lines. **(D)** Kaplan-Meier analysis of overall survival of 515 RCC patients in TCGA-KIRC stratified by high and low FAM13A-AS1 expression.

FAM13-AS1 is a long non-coding RNA that was recently identified as a prognostic biomarker for thyroid cancer (Rao et al., 2020). However, its role in RCC has not yet been studied. We found that the expression level of FAM13-AS1 was significantly elevated in RCC by analyzing the expression profiles of 539 RCC samples and 72 normal tissue samples from the TCGA-KIRC database, indicated by the scatter plot generated from the whole dataset (*p* < 0.001, Figure 1A) and 72 paired tissue samples (*p* = 0.0083, Figure 1A). To further support this finding, we used RT-qPCR to measure the expression level of FAM13-AS1 in an independent RCC cohort, containing 40 RCC patients whose tumor and paracancerous tissues were sampled. Compared to normal renal tissues, FAM13-AS1 expression was significantly increased in tumor tissues, with *p* < 0.01 and *p* = 0.0012 for overall and paired scatterplots, respectively (Figure 1B). In addition, we tested FAM13-AS1 expression in multiple kidney cell lines. Three different human kidney cancer cell lines, 786-O, SN12-PM6, and Caki-1, showed significantly higher expression of FAM13A-AS1 than the normal renal epithelial cell line HK-2. No difference in FAM13A-AS1 expression was observed in the A-498 cells (Figure 1C). This evidence suggests that FAM13A-AS1 may be universally overexpressed in different types of RCC. Thus, we investigated whether the expression level of FAM13A-AS1 correlates with any clinical outcome. Kaplan-Meier survival analysis showed that patients with low expression of FAM13A-AS1 had significantly better overall survival rates (*p* = 0.0007) (Figure 1D), suggesting that FAM13A-AS1 could be a promising prognostic marker for RCC.

### FAM13A-AS1 Promotes Cell Proliferation, Migration, and Invasion in Kidney Cancer Cell Lines

**FIGURE 2:**
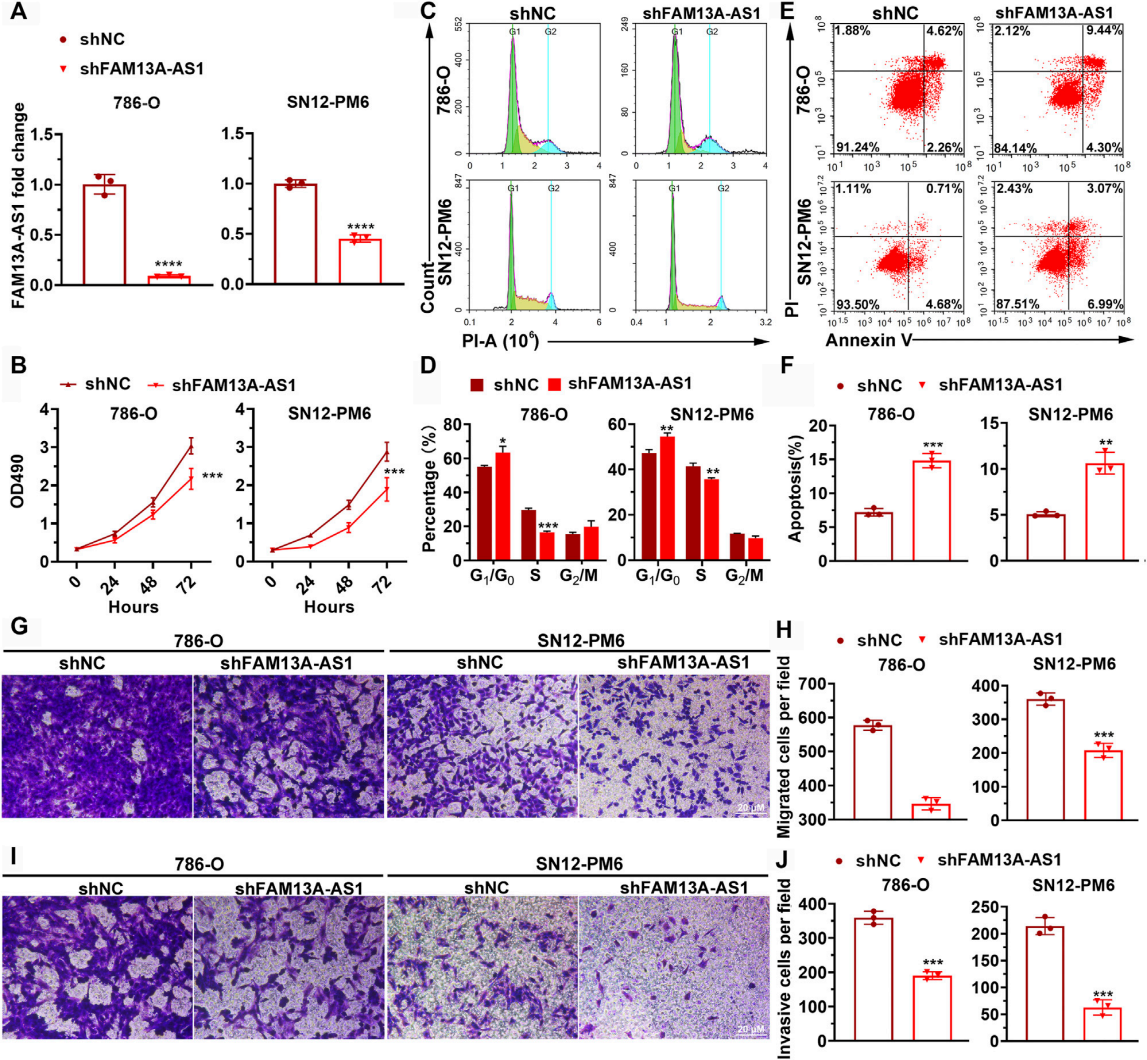
FAM13A-AS1 promotes RCC cell proliferation, migration, and invasion. **(A)** Bar plot showing the knockdown efficiencies of shRNA targeting FAM13A-AS1. Expression levels were measured by RT-qPCR and calibrated using the expression level of 18S rRNA. Fold changes in expression were then computed against the scrambled control. **(B)** MTT assay of RCC cells transfected with shFAM13A-AS1 or scrambled control. **(C,D)** Representative histograms **(C)** and quantification **(D)** of cell cycle distribution of RCC cells transfected with shFAM13A-AS1 or scrambled control. *X* and *Y* axes in **(C)** denote DNA content and cell number, respectively. **(E,F)** Dot plot **(E)** and bar chart **(F)** of Annexin V/PI two-parameter flow cytometry analysis of RCC cells transfected with shFAM13A-AS1 or scrambled control. Bar plot **(F)** shows the percentages of cells that underwent apoptosis. **(G,H)** Representative images **(G)** and quantification **(H)** of the Transwell migration assay in RCC cells transfected with shFAM13A-AS1 or scrambled control. **(I,J)** Representative images **(I)** and quantification **(J)** of the matrigel invasion assay in RCC cells transfected with shFAM13A-AS1 or scrambled control. Data represent the mean ± SD. **: *p* < 0.01; ***: *p* < 0.001; ****: *p* < 0.0001.

Many long non-coding RNAs have been reported to promote tumorigenesis and the progression of various types of cancers ^3^ . To explore whether FAM13A-AS1 has similar functions in RCC, we examined cell proliferation, migration, and invasion in both 786-O and SN12-PM6 cells transfected with shRNA against FAM13A-AS1. The knockdown efficiency of shRNA was confirmed by RT-qPCR analysis (Figure 2A). MTT assays indicated that shRNA knockdown of FAM13A-AS1 significantly impaired cell growth and proliferation rate, compared to cells transfected with control shRNA (Figure 2B). To explore the possible mechanisms underlying the increase in cell proliferation rates, we performed flow cytometry to examine the cell cycle of 786-O and SN12-PM6 cells transfected with shFAM13A-AS1 or scramble control. We found that reduced FAM13A-AS1 expression was associated with cell cycle arrest at the G_0_/G_1_ stage (Figures 2C,D). In particular, the mean percentage of cells in the G_0_/G_1_ phase increased by 10% in the FAM13A-AS1 knockdown cell line compared to the control. Consistently, Annexin V staining indicated that FAM13A-AS1 inhibition promoted cell apoptosis (Figures 2E,F). The percentage of cells undergoing apoptosis was significantly higher in cells transfected with shFAM13A-AS1 than in cells transfected with scrambled control. Moreover, we found that cells with reduced FAM13A-AS1 expression displayed significantly less migration and invasion activities, as revealed by the Transwell assay (Figures 2G,H) and matrigel invasion assays, respectively (Figures 2I,J). Collectively, these results indicate that FAM13A-AS1 is a potential oncogenic lncRNA in RCC.

### FAM13A-AS1 Attenuates miR-141-3p Function by Acting as a ceRNA

**FIGURE 3:**
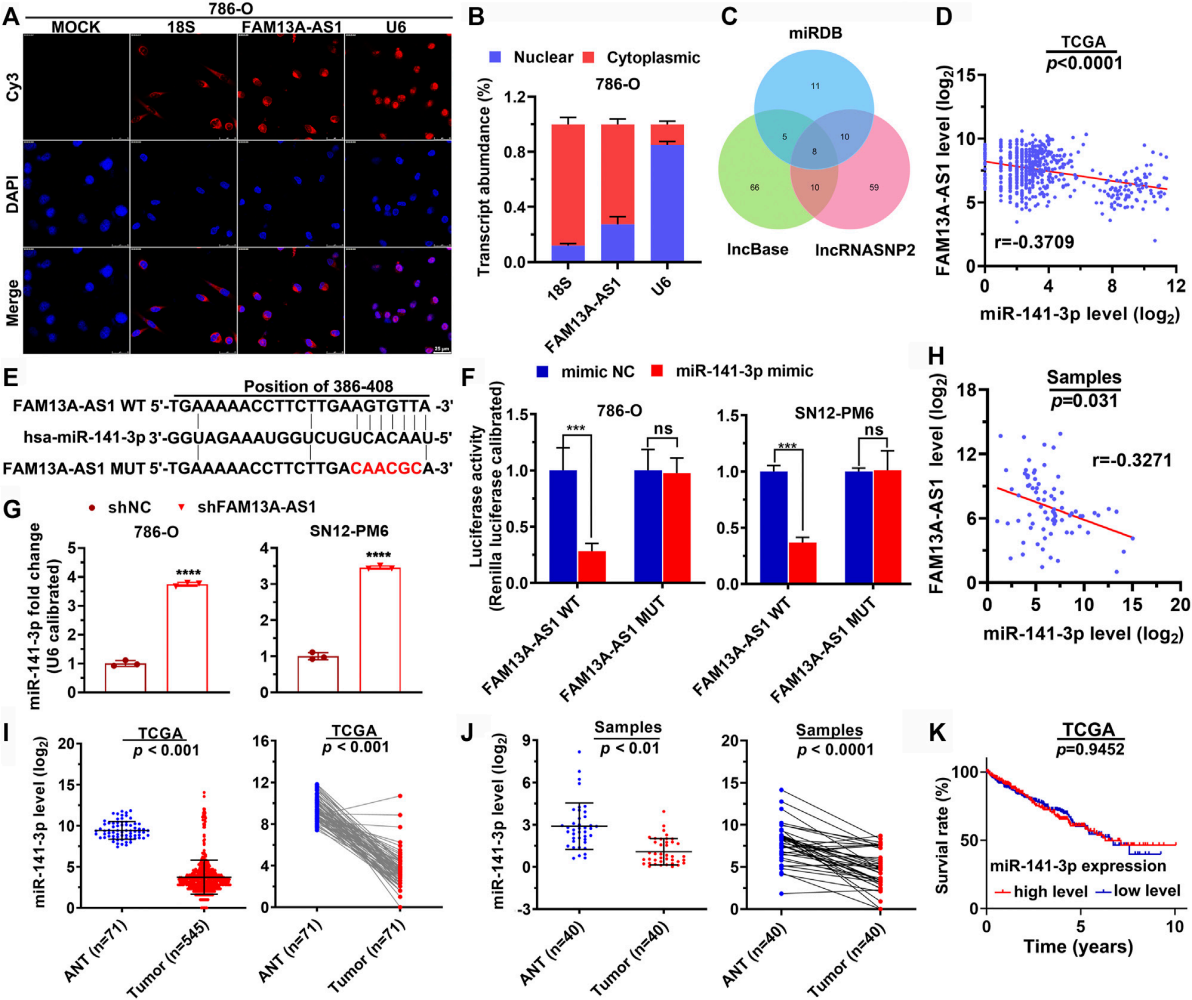
FAM13A-AS1 functions as a ceRNA that competes for miR-141-3p. **(A,B)** Fish assay **(A)** and RT-qPCR **(B)** showing the subcellular expression (nucleus and cytoplasm) of FAM13A-AS1 in RCC cells, compared with 18S and U6 as the endogenous controls. **(C)** Venn diagram showing the intersection between computationally identified FAM13A-AS1 interacting miRNAs. **(D)** Scatter plot showing the co-expression pattern between miR-141-3p (Y-axis) and FAM13A-AS1 (*X*-axis) in TCGA. **(E)** Schematic showing the predicted miR-141-3p binding sites in FAM13A-AS1 wild-type transcript, and the FAM13A-AS1 mutant escaping the binding of miR-141-3p. The red nucleotides represent mutations on target sites. **(F)** Bar plot showing the luciferase activities in RCC cells co-transfected with wild-type (WT) or mutant (MUT) FAM13A-AS1 plasmid together with miR-141-3p mimic or negative control (NC). **(G)** Bar plots showing the miR-141-3p expression in RCC cells transfected with shFAM13A-AS1 or negative control (NC). Expression levels were measured using RT-qPCR. **(H)** Scatter plot showing the co-expression pattern between miR-141-3p (Y-axis) and FAM13A-AS1 (*X*-axis) in and our own cohorts. **(I,J)** Scatter plots showing the miR-141-3p expression in normal and tumor tissues of RCC patient from TCGA **(H)** and our own **(I)** cohorts. **(K)** Kaplan-Meier analysis of the overall survival rate of RCC patients stratified by the expression of miR-141-3p from TCGA cohort. Data represent the mean ± SD. ***: *p* < 0.001; ****: *p* < 0.0001.

To investigate how FAM13A-AS1 exerts the function, we first explored its subcellular locations in 786-O kidney cancer cells by FISH assay. FAM13A-AS1 transcripts were recognized and labeled by cy3, which mainly remained in the cytoplasm, almost exclusively from DAPI staining (Figure 3A). These results were further confirmed by RT-qPCR using subcellular fractionation. This analysis showed that 73% of FAM13A-AS1 transcripts resided in the cytoplasm pool, whereas only 27% were retained inside the nucleus (Figure 3B). Therefore, we hypothesized that FAM13A-AS1 might function as a ceRNA to regulate miRNA species. Using bioinformatics tools, we predicted potential interacting miRNAs from three different databases (Figure 3C). A total of 188 miRNA targets could interact with FAM13A-AS1, and five overlapped across all three databases. We further examined their expression levels in RCC by analyzing the published miRNA expression profiles. Among the five shared miRNA targets, we found that one miRNA, miR-141-3p, was negatively correlated with FAM13A-AS1 (Figure 3D, *p* < 0.0001). These findings suggested us that FAM13A-AS1 may interact with miR-141-3p. Using a luciferase reporter assay, we revealed that overexpression of miR-141-3p led to decreased luciferase activity of the wild-type FAM13A-AS1 reporter gene (Figures 3E,F). In contrast, the miR-141-3p mimic did not alter the luciferase activity of the FAM13A-AS1 mutant carrying mutations at the putative miRNA-binding site. Moreover, knockdown of FAM13A-AS1 could, in turn, elevate the expression level of miR-141-3p in both 786-O and SN12-PM6 cells (Figure 3G). In addition, the negative correlation between the expression of FAM13A-AS1 and miR-141-3p was also confirmed in our RCC patient cohort (Figure 3H, *p* = 0.031). These findings confirmed that FAM13A-AS1 serve as a ceRNA for miR-141-3p. Finally, we assessed the expression of miR-141-3p in patients from the TCGA and RCC cohorts. Expression of miR-141-3p was significantly repressed in RCC tumor samples compared to that in normal paracancerous tissues (Figures 3I,J). However, miR-141-3p alone is not a good estimator for overall patient survival, as the Kaplan-Meier survival analysis showed no difference in the overall survival between RCC patients classified into high and low miR-141-3p expression (Figure 3K). This may attribute to the limited number of samples. Taken together, these findings suggest that FAM13A-AS1 binds to miR-141-3p as a decoy to sequester the miRNA away from its target.

**FIGURE 4:**
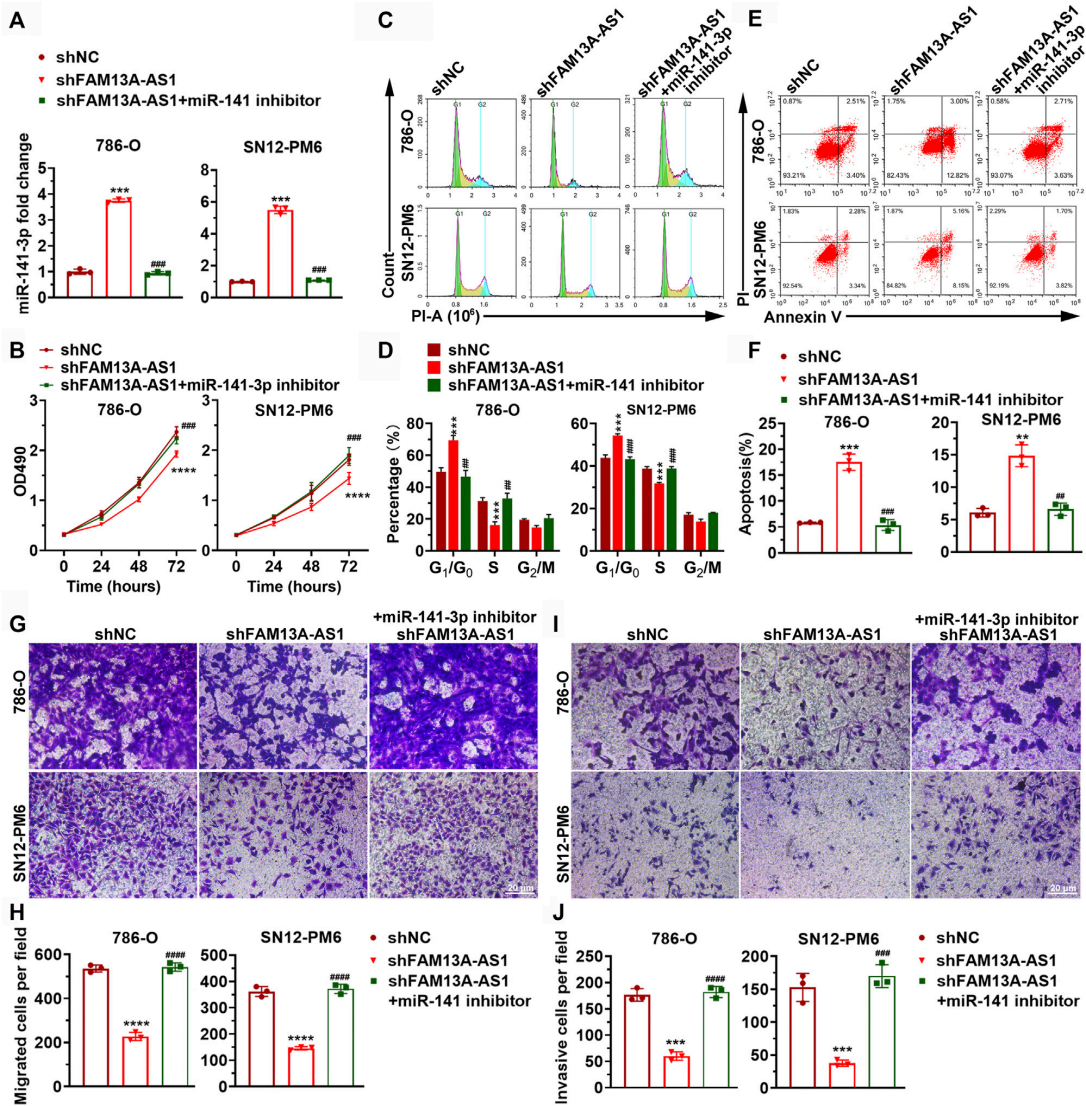
miR-141-3p inhibits kidney cancer cell proliferation, migration, and invasion. **(A)** Bar plot showing the expression level of miR-141-3p in RCC cells co-transfected with shFAM13A-AS1 or scrambled control treated with either miR-141-3p. **(B)** MTT assay of RCC cells transfected with shFAM13A-AS1 treated with either miR-141-3p inhibitor or negative controls. **(C,D)** Representative images **(C)** and quantification **(D)** of cell proliferation assay in RCC cells. Edu-positive cells: green. Cell nuclei: blue. **(C,D)** Representative histograms **(C)** and quantification **(D)** of cell cycle distribution of RCC cells. *X* and *Y* axes **(C)** denote DNA content and cell number, respectively. Bar plot **(D)** shows the percentages of cells at different stages during the cell cycle. **(E,F)** Representative dot plots **(E)** and quantification **(F)** of Annexin V/PI two-parameter flow cytometry analysis of RCC cells. Bar plot (lower) shows the percentages of cells that undergo apoptosis. The RCC cell lines were transfected with shFAM13A-AS1 or scrambled control and treated with miR-141-3p inhibitor. **(G,H)** Representative images **(G)** and quantification **(H)** of the transwell migration assay in RCC cells transfected or treated with miR-141-3p inhibitor. **(I,J)** Representative images **(I)** and quantification **(J)** of the matrigel invasion assay in the above cells. Data represent the mean ± SD. ***: *p* < 0.001; ****: *p* < 0.0001.

To further establish the hypothesis that the carcinogenic effect of FAM13A-AS1 is mediated by negative regulation of miR-141-3p, we introduced miR-141-3p mimic or miR-141-3p inhibitor in kidney cancer cells transfected with shFAM13A-AS1 (Figure 4A). Consistent with our previous findings, silencing of FAM13A-AS1 led to elevated expression of miR-141-3p. In addition, we found that the miR-141-3p inhibitor fully rescued the phenotypic effects resulting from FAM13A-AS1 silencing. In particular, inhibition of miR-141-3p function promoted cell proliferation (Figures 4B–D), cell migration (Figures 4G,H), and cell invasion (Figures 4I,J). The miR-141-3p knockdown mediated increase in cell proliferation was associated with increased DNA synthesis and the release from the cell cycle arrest at the G_0_/G_1_ phase, as revealed by the EdU cell proliferation assay and flow cytometry analysis, respectively (Figures 4B–D). Similarly, Annexin V staining showed fewer cells underwent apoptosis when miR-141-3p silencing was introduced in RCC cells transfected with shFAM13A-AS1 (Figures 4E,F). Based on our findings, we conclude that FAM13A-AS1 functions as an oncogene in RCC, partially by negatively regulating the expression of miR-141-3p.

### FAM13A-AS1 Sponges miR-141-3p to Upregulate Its Target NEK6

**FIGURE 5:**
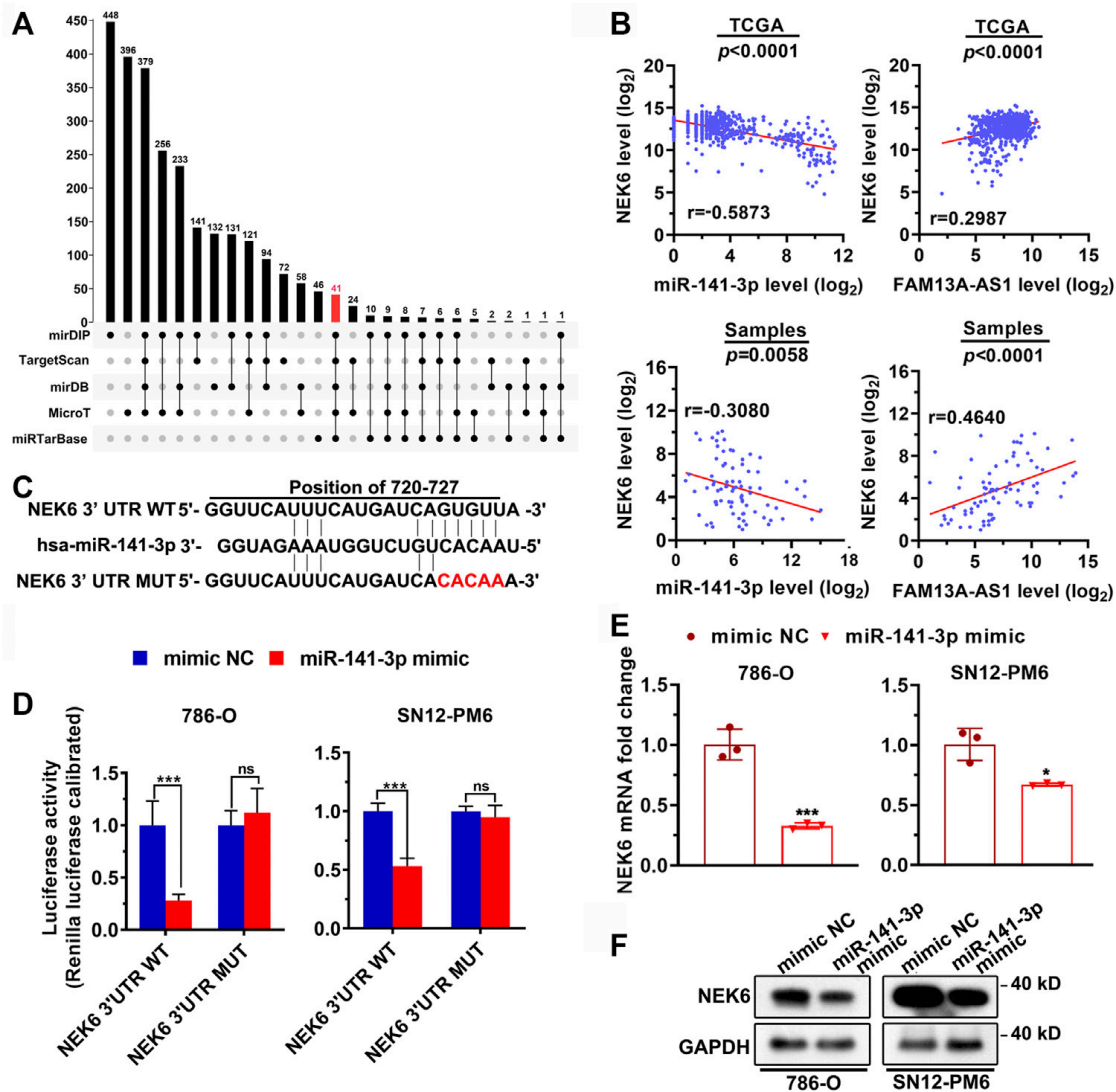
FAM13A-AS1 sponges miR-141-3p to upregulate its target NEK6. **(A)** Upset plot showing the intersections between five sets of computationally identified miR-141-3p targeting genes. **(B)** Correlation between NEK6 and miR-141-3p or FAM13A-AS1 expression in RCC from TCGA and our own cohorts. **(C)** Schematic showing the predicted miR-141-3p binding sites in 3′UTR of NEK6 wild-type and mutant escaping the binding of miR-141-3p. The red nucleotides represent mutations on target sites. **(D)** Bar plot showing the luciferase activities in 786-O and SN2-PM6 cells co-transfected with wild-type (WT) or mutant (MUT) NEK6 plasmid together with miR-141-3p mimic or negative control (NC). **(E,F)** Bar plots **(E)** and blotting image **(F)** showing the mRNA and protein levels of NEK6 expression in 786-O and SN2-PM6 cells transfected with miR-141-3p mimic, or negative controls (NC) from RT-qPCR and western blot analysis. Data represent the mean ± SD. **: *p* < 0.01; ***: *p* < 0.001; ****: *p* < 0.0001.

To identify the genes regulated by miR-141-3p, we first predicted the target genes of miR-141-3p using five different computational software packages (Figure 5A). mirDIP recovered the largest number of target hits, a total of 448 predicted target genes, whereas miRTarbase only reported 48 potential targets. Notably, there were 41 common targets predicted by all the software. To further identify the most relevant gene targets, we performed a correlation analysis between the expression level of each candidate target and miR-141-3p or FAM13A-AS1 in tumor samples from patients with RCC. Among all the tested mRNAs, NEK6, a gene that encodes a kinase required for progression through the metaphase portion of mitosis, is of particular interest. We found that the expression level of NEK6 was significantly positively correlated with FAM13A-AS1 in the TCGA cohort (Figure 5B, *p* < 0.0001). In addition, a strong negative correlation was observed between NEK6 and miR-141-3p expression (*p* < 0.0001). RT-qPCR confirmed the results in 40 patients with RCC. These results together suggest that NEK6 is a direct target of miR-141-3p. Consistent with this, using a luciferase reporter assay, we showed that overexpression of miR-141-3p significantly inhibited the luciferase activity of RCC cells transfected with wildtype NEK6 3’ -UTR reporter. In contrast, no repression of luciferase activity was observed in cells transfected with the mutant reporter (Figures 5C,D). Next, we investigated whether endogenous expression of NEK6 is affected by the miR-141-3p mimic. RT-qPCR and western blotting revealed that both the mRNA and protein levels of NEK6 were reduced with miR-141-3p overexpression than the mimic negative control (Figures 5E,F). In summary, we identified NEK6 as a gene target of miR-141-39 in RCC.

Expression of NEK6 is elevated and associated with poor clinical outcome in RCC patients.

**FIGURE 6:**
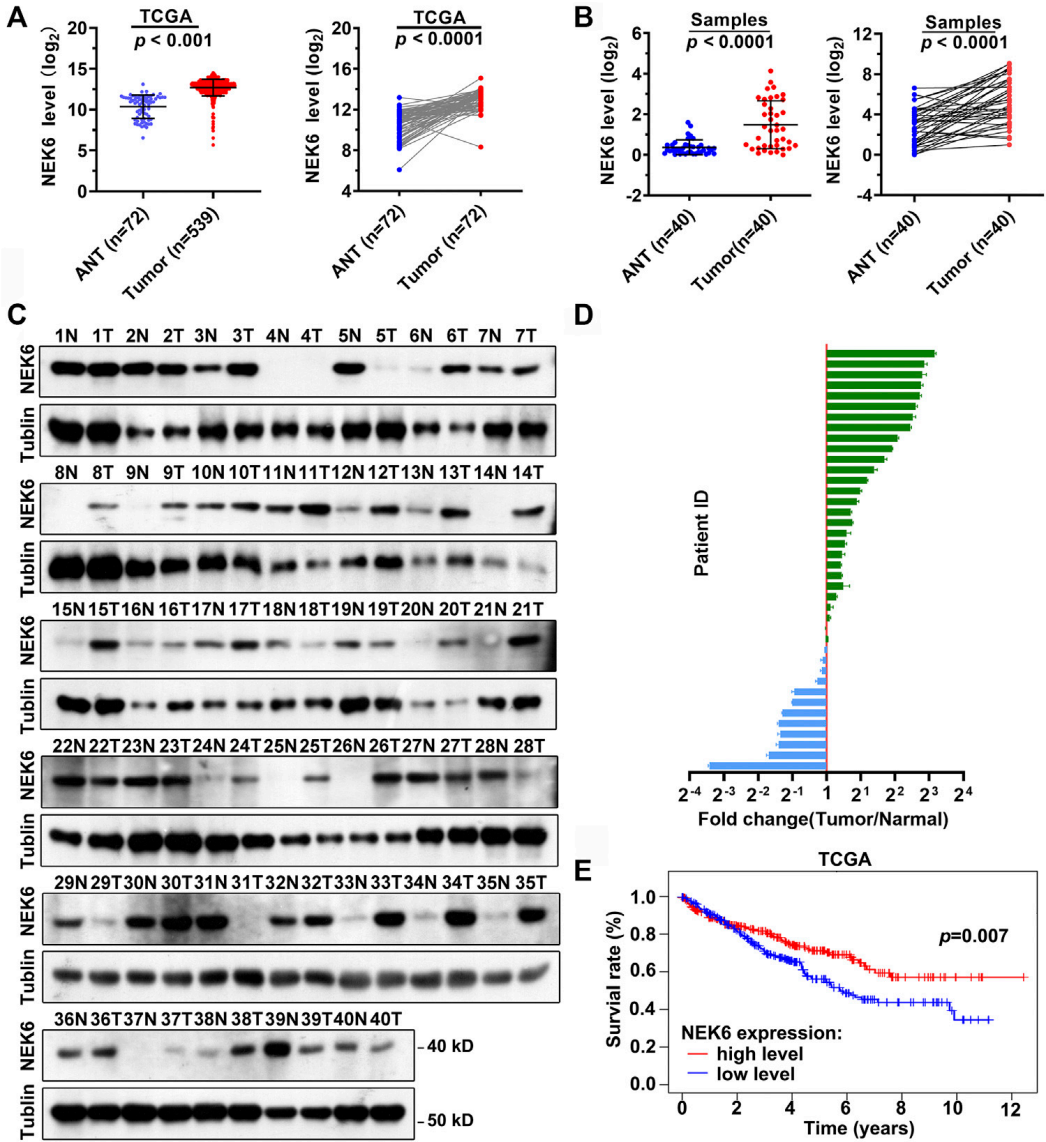
Elevated expression level of NEK6 in RCC. **(A)** Scatter plots showing the expression of NEK6 in RCC (*n* = 539) and normal tissues (*n* = 72) from TCGA cohort. **(B)** Scatter plots showing the expression levels of NEK6 in RCC (*n* = 40) and normal tissues (*n* = 40) from our cohort. **(C,D)** Blotting images **(C)** and quantification **(D)** of protein level of NEK6 in RCC and normal tissues from our cohort. **(E)** Kaplan-Meier analysis of the overall survival rate of RCC patients stratified by the expression of NEK6 from TCGA cohort.

The NEK6 transcript has been reported to be significantly upregulated in hepatocellular carcinoma. However, it is not clear how NEK6 functions in RCC. By analyzing the transcriptome of the TCGA cohort, we noticed that the expression level of NEK6 in RCC was significantly increased in tumor samples, compared with the adjacent normal tissues (Figure 6A). A similar observation was obtained from 40 additional RCC patients. RT-qPCR indicated that NEK6 expression was significantly elevated in RCC (Figure 6B). We further determined the protein level of NEK6 in all RCC biopsies. Of the 40 patients, 12 displayed decreased protein levels of NEK6 in tumors when normalized to their paired normal tissue. The rest had an elevated NEK6, with the highest showing more than an eight-fold change (Figures 6C,D). Surprisingly, Kaplan-Meier survival analysis suggested that NEK6 is a favorable estimator for the overall OS of RCC patients from the TCGA cohort. We found that patients with high NEK6 expression showed better survival rates than those with low NEK6 expression.

### FAM13A-AS1 Exerts Oncogenic Effect by Regulating NEK6

**FIGURE 7:**
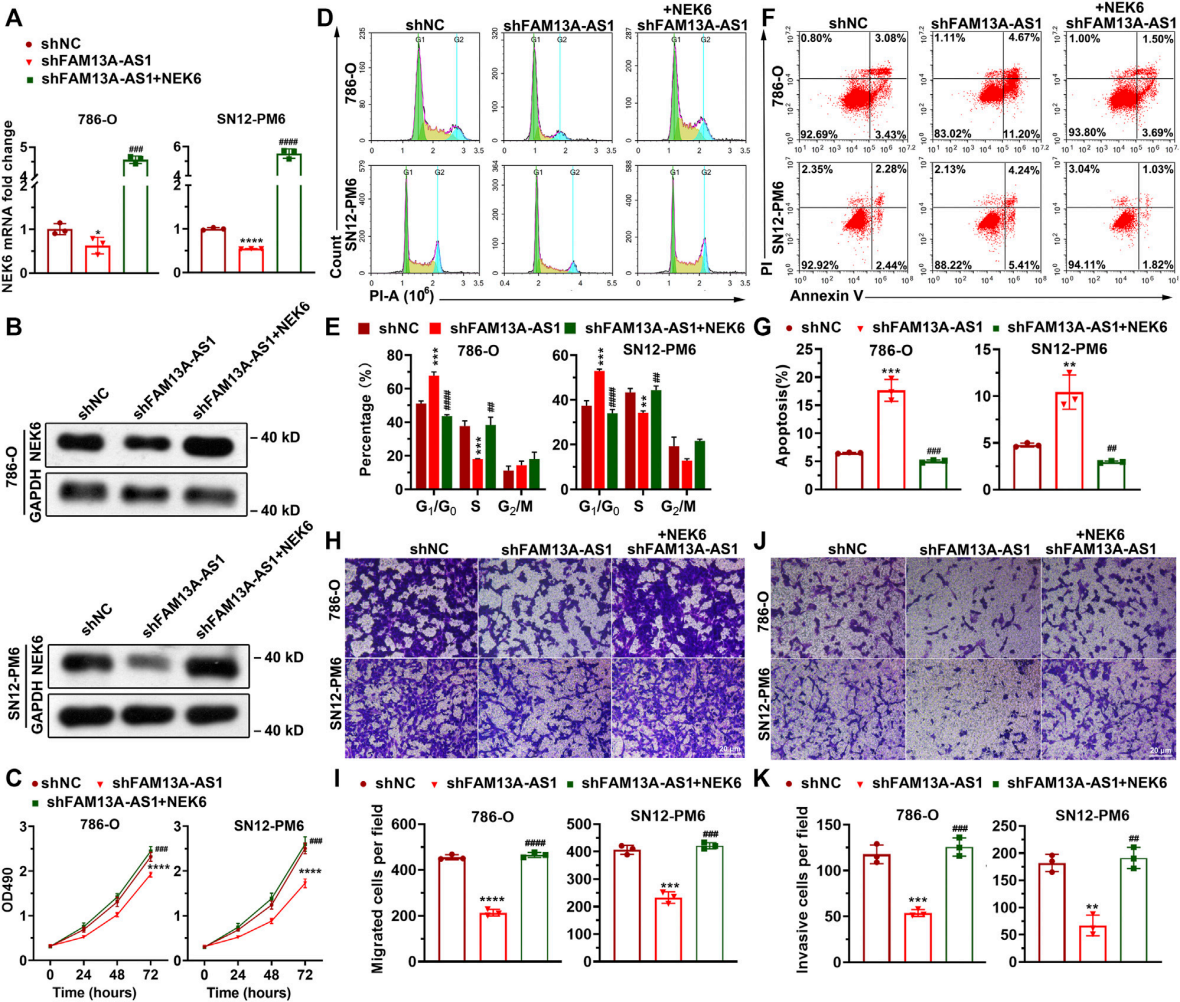
NEK6 is responsible for FAM13A-AS1-mediated cell proliferation, migration, and invasion. **(A)** Bar plots showing the expression of NEK6 in 786-O and SN12-PM6 cells transfected with shFAM13A-AS1 or negative control (shNC), together with empty vector or NEK6 overexpression plasmid. Expression levels were measured by RT-qPCR. Fold changes of expression were then computed against the scrambled control. **(B)** Blotting images showing the protein level of NEK6 in the above cells. **(C)** MTT assay of 786-O and SN12-PM6 cells transfected with shFAM13A-AS1 or negative control (shNC), together with empty vector or NEK6 overexpression plasmid. **(D–K)** Representative images and quantifications of the flow cytometry analysis for cell cycle distribution **(D,E)**, Annexin V/PI two-parameter flow cytometry analysis **(F,G)**, transwell migration assay **(H,I)**, and the matrigel invasion assay **(G,K)** of 786-O and SN12-PM6 cells transfected with shFAM13A-AS1 or negative control (shNC), together with empty vector or NEK6 overexpression plasmid. Data represent the mean ± SD. *: *p* < 0.05; **: *p* < 0.01; ***: *p* < 0.001; ****: *p* < 0.0001.

To further establish our hypothesis that FAM13A-AS1 functions by negatively regulating miR-326, we used shRNA to knockdown FAM13A-AS1 in RCC cells, which resulted in a significant reduction in NEK6 RNA and protein levels, resembling the effect of transfection with miR-141-3p mimic (Figures 7A,B). The downregulation of NEK6 expression was reversed, and the expression level was comparable to that in RCC cells transfected with a scrambled control, with miR-141-3p inhibitor treatment. Overexpression of NEK6 restored its mRNA and protein levels (Figures 7A,B). In addition, we found that inhibition of cell proliferation (Figures 7C–E), apoptosis (Figures 7F,G), migration (Figures 7H,I), and invasion (Figures 7J,K) caused by the FAM13A-AS1 knockdown was rescued by NEK6 overexpression. These results together confirmed that FAM13A-AS1 enhances cell proliferation, migration, and invasion in a NEK6-dependent manner.

### FAM13A-AS1 Promotes RCC Tumorigenesis *in Vivo*

**FIGURE 8:**
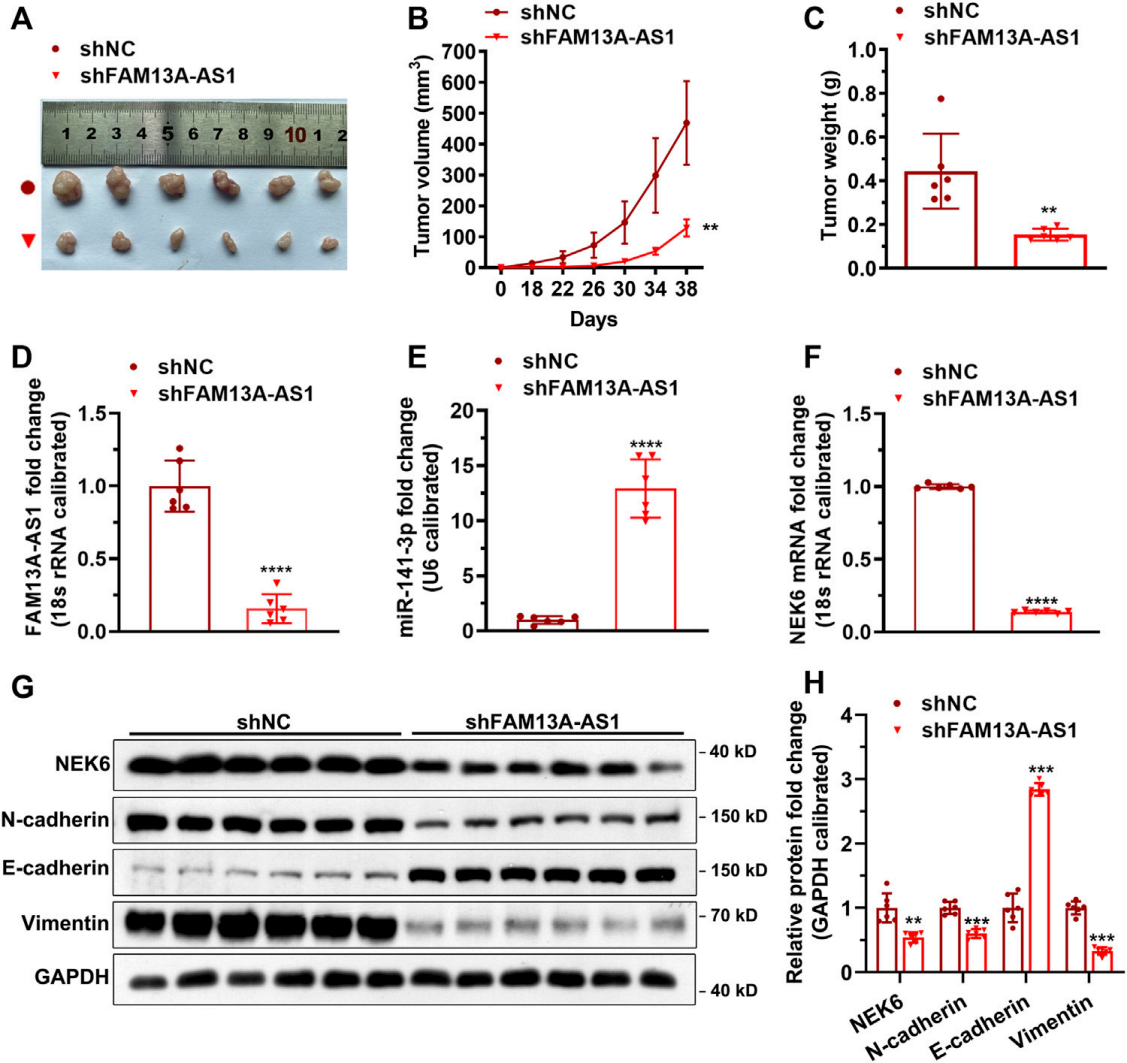
FAM13A-AS1 promotes tumorigenesis *in vivo*. **(A-C)** Xenograft tumor model was constructed by transplanting the above 786-O cells into nude mice with representative images **(A)**, measurement of tumor volume **(B)**, and tumor weight **(C)**. **(D-F)** Expression of FAM13A-AS1 **(D)**, miR-141-3p**(E)**, and NEK6 **(F)** in the tumors revealed by RT-qPCR, calibrated using the expression level of 18S rRNA or U6. Fold changes of expression were then computed against the scrambled control. **(G,H)** Blotting images **(G)** and quantification **(H)** of protein levels in the tumor tissues (*n* = 6). Data represent the mean ± SD. **: *p* < 0.01; ***: *p* < 0.001; ****: *p* < 0.0001.

We have demonstrated that FAM13A-AS1 exerts its carcinogenic function by forming the lncRNA/miRNA/target axis with miR-141-3p and NEK6 in kidney cancer cell lines. To confirm that a similar mechanism exists *in vivo,* we generated a transgenic mouse model carrying shFAM13A-AS1 to induce FAM13A-AS1 silencing (Figure 8A). Our results showed that FAM13A-AS1 dramatically inhibited tumor growth (Figures 8B,C). A significant reduction in both tumor volume and weight was observed in mice with FAM13A-AS1 silencing. The tumor volume decreased from 580 to 160 mm^2^, and the tumor weight was reduced to only 35% on day 38 in shFAM13A-AS1 transgenic mice compared to scrambled shRNA. The decreased expression of FAM13A-AS1 was confirmed in the tumor tissues, which was accompanied by an increased level of miR-141-3p (Figures 8D,E). Moreover, FAM13A-AS1 knockdown led to the downregulation of NEK6 *in vivo* (Figures 8F–H). Both the mRNA (Figure 8F) and protein levels (Figures 8G,H) of NEK6 were reduced in shFAM13A-AS1 tumors. In addition, FAM13A-AS1 deficiency regulated the protein levels of EMT markers such as N-cadherin, E-cadherin, and vimentin (Figures 8G,H). Thus, our data suggest that FAM13A-AS1 promotes the tumorigenesis of RCC *in vivo*.

## Discussion

RCC is one of the most common cancers worldwide. In contrast to breast cancer, which is frequently diagnosed at an early stage and is widely treated by conservative surgery, RCC has a high incidence of metastasis ^13^ . Its incidence is also increasing every year, especially among the elderly. The mainstay of treatment for metastatic RCC is androgen deprivation therapy (ADT), which reduces the levels of androgens in the body ^14^ . In the initial stages of ADT, most patients experienced improved symptoms and increased quality of life. However, ADT is associated with several adverse effects in advanced metastatic RCC. As a result, there is a need for novel and effective approaches to diagnose and treat RCC. In the present study, we identified FAM13A-AS1 as a potential biomarker for RCC. FAM13A-AS1 is an lncRNA that has been associated with non-small cell lung carcinoma (NSCLC) (Yu et al., 2015). Distinct expression of FAM13A-AS1 has been reported in NSCLC patients. In addition, Sun *et al.* built a five-lncRNA prognostic signature that contains FAM13A-AS1 to predict the survival outcome of bladder urothelial carcinoma patients (Sun et al., 2020). However, its role in RCC has never been studied before. We found that FAM13A-AS1 was significantly upregulated in RCC tissues and positively correlated with adverse clinical outcomes. We further demonstrated that FAM13A-AS1 is a ceRNA of miR-141-3p and that the expression levels of these two transcripts have a strong negative correlation in RCC patients. Moreover, we established that FAM13A-AS1 promotes the proliferation, migration, and invasion of RCC cells using overexpression and knockdown experiments. These results suggest that FAM13A-AS1 is involved in the tumorigenesis of RCC.

MicroRNAs (miRNAs) are endogenous, small non-coding RNAs that regulate gene expression by binding to the 3′-UTRs of their mRNA targets (Bartel, 2018). In the past decades, accumulating evidence has shown that aberrant miRNAs are associated with carcinogenesis (Peng and Croce, 2016). As such, miR-141-3p has been implicated in various malignant tumors. miR-141-3p was first identified as a negative regulator in stromal stem cells to regulate cell proliferation by targeting CDC25A (Qiu and Kassem, 2014). More recently, the potential oncogenic role of miR-141-3p in prostate cancer was revealed by Li et ai. (Li et al., 2017). The authors found that miR-141-3p

miR-141-3p promoted the stemness of prostate cancer cells by negatively regulating Krüppel-l-like factor 9. Additional evidence revealed that miR-141-3p could inhibit the expression of GLI2, downregulating parathyroid hormone-related protein 1 and promoting apoptosis in osteosarcoma cells (Wang et al., 2018). In this study, we showed that miR-141-3p was also downregulated in RCC. Our findings showed the significance of the interaction between FAM13A-AS1 and miR-141-3p in RCC. Treatment with miR-141-3p inhibitor fully rescued the suppression of cell proliferation, migration, and invasion resulting from FAM13A-AS1 silencing.

NEK6 is a protein kinase required for progression through the metaphase portion of mitosis. It is involved in the control of mitotic spindle formation, acting together in a mitotic kinase cascade with other NIMA-family kinases (O’Regan and Fry, 2009; Meirelles et al., 2011). Inhibition of the expression of NEK6 can lead to apoptosis. This protein has been implicated in the enhancement of tumorigenesis by suppressing tumor cell senescence. It was recently shown that NEK6 expression was significantly upregulated in hepatocellular carcinoma (Chen et al., 2006). Earlier studies have also demonstrated elevated expression of NEK6 in multiple malignancies, such as breast cancer, colorectal cancer, lung cancer, and laryngeal cancer (Capra et al., 2006). Similar mechanisms have been uncovered in advanced gastric cancers by Takeno et al. (Takeno et al., 2008). Using bioinformatics tools, we predicted NEK6 as a potential target of miR-141-3p, which was confirmed by a luciferase reporter assay. Furthermore, we showed that overexpression of miR-141-3p suppressed the expression of NEK6 at both the mRNA and protein levels. Finally, we showed that overexpression of NEK6 rescued the growth inhibition phenotype induced by FAM13A-AS1 knockdown. Collectively, these results suggest that FAM13A-AS1 promotes tumor growth by competing miR-141-3p with NEK6.

It should be noted that there are also several limitations for this study. For example, investigation the effect of FAM13A-AS1 overexpression on normal cells may further confirm our conclusion, which will be done in the next work. Moreover, the pulmonary metastatic model should be established to evaluate the effect of FAM13A SA1/miR-141/NEK6 axis on the metastatic ability of RCC cells.

In conclusion, FAM13A-AS1 serve as an oncogene through miR-141-3p/NEK6 axis and may be a potential target for RCC treatment.

## Data Availability

The original contributions presented in the study are included in the article/Supplementary Material, further inquiries can be directed to the corresponding author.
