# Supplementary material for: LncRNA FAM13A-AS1 Promotes Renal Carcinoma Tumorigenesis Through Sponging miR-141-3p to Upregulate NEK6 Expression
Source: Front Mol Biosci. 2022 Mar 23;9:738711. doi: 10.3389/fmolb.2022.738711 (PMC8984162; doi:10.3389/fmolb.2022.738711)
Supplement: Supplementary file 1 [file DataSheet1.DOCX]

Download link for original data:

<https://www.jianguoyun.com/p/DZL96csQyKbVCRi-h4EE>

<https://www.jianguoyun.com/p/DW0h_jEQyKbVCRjBh4EE>

<https://www.jianguoyun.com/p/DWIEdecQyKbVCRjCh4EE>

<https://www.jianguoyun.com/p/DWrVIaQQyKbVCRjDh4EE>

<https://www.jianguoyun.com/p/DZu7l9cQyKbVCRjEh4EE>

<https://www.jianguoyun.com/p/DWS-qkwQyKbVCRjFh4EE>

<https://www.jianguoyun.com/p/DcM7tZsQyKbVCRjGh4EE>

<https://www.jianguoyun.com/p/DUPDUIwQyKbVCRjHh4EE>

We cannot set the sharing scope for any download links to "Anyone"，but ”all people who register “jianguoyun”.
